# Spatiotemporal patterns of lung disease in China before 2019: A brief analysis of two nationally representative surveys

**DOI:** 10.1371/journal.pone.0278031

**Published:** 2022-11-21

**Authors:** Andrew Francis-Tan, Xueqing Wang

**Affiliations:** 1 Lee Kuan Yew School of Public Policy, National University of Singapore, Singapore, Singapore; 2 Office of Population Research, School of Public and International Affairs, Princeton University, Princeton, New Jersey, United States of America; China National Center for Food Safety Risk Assessment, CHINA

## Abstract

Little is publicly known about the conditions surrounding the emergence of COVID in China. Using two nationally representative datasets, the China Family Panel Studies (CFPS) and the China Health and Retirement Longitudinal Study (CHARLS), we engage in a descriptive analysis of spatiotemporal patterns of lung and other diseases before 2019. In both datasets, the incidence of lung disease in 2018 was elevated in Hubei province relative to other provinces. The incidence of psychiatric and nervous system disease was elevated as well. Overall, the evidence is consistent with many possible explanations. One conjecture is that there was an outbreak of influenza in central China, which implies the conditions that increased the susceptibility to influenza also facilitated the later spread of COVID. Another conjecture, though less likely, is that COVID was circulating at low levels in the population in central China during 2018. This study calls for more investigation to understand the conditions surrounding the emergence of COVID.

## Introduction

For over two years, severe acute respiratory syndrome coronavirus 2 (SARS-CoV-2), the virus that causes COVID-19, has swept the globe. The first cases of COVID in humans were identified in December 2019 in the city of Wuhan, the capital of Hubei province. Many see the Huanan Seafood Market in Wuhan as "ground zero" of the pandemic. Evidence on the importance of the Huanan Seafood Market is mixed. Recent studies affirm the possibility that human spread began at the market [1,2]. However, studies from Spain, Italy, France, Brazil and the U.S. retrospectively found SARS-CoV-2 in blood, sewage, and other samples collected in 2019, suggesting that COVID circulated earlier than thought [3, page 82].

A report jointly written by the WHO and China contains most of the recognized evidence regarding the origin of COVID [3]. The report is most confident about the zoonotic origins of SARS-CoV-2. Genome analysis suggests that a bat population is likely the ecological source of the virus [4]. Nevertheless, the epidemiological evidence is relatively thin. Data from all-cause mortality and pneumonia mortality from Wuhan and Hubei in 2019 and early 2020 exhibit a pattern suggesting that COVID emerged in Wuhan by late 2019 [3,5]. Mortality data provides little evidence of COVID transmission prior to December 2019, but the report notes the evidence does not exclude the possibility that transmission was occurring at a low level [3].

Data from influenza-like illness (ILI) and severe acute respiratory infection (SARI) provide no evidence of COVID transmission in the months preceding the outbreak in December 2019. In particular, patterns of ILI were similar in 2019 between Wuhan and other cities in Hubei and between Hubei and provinces surrounding Hubei. There was a notable spike in ILI and SARI at the end of 2019, but this was mostly attributable to children with influenza [3]. Nevertheless, the report’s epidemiological data come from a limited number of sentinel hospitals (i.e., two in Wuhan collect information on ILI, and one in Hubei collects information on SARI). The analysis relies on comparisons of places within central China, so it would be challenging to detect COVID transmission if the outbreak were occurring throughout the region.

Thus, relatively little is publicly known about the conditions surrounding the emergence of COVID. The objective of this paper is to describe spatiotemporal patterns of lung disease in China before 2019. The paper uses two nationally representative surveys: the China Family Panel Studies (CFPS), which covers the general adult population, and the China Health and Retirement Longitudinal Study (CHARLS), which covers the older adult population. The data are far from ideal but still may yield clues to guide future research on the reasons COVID emerged where and when it did.

## Methods

Ethics approval for this study was given by the National University of Singapore IRB. Stata version 15 was used to perform the statistical analyses.

### CFPS

The China Family Panel Studies (CFPS) is a large-scale longitudinal survey focused on family and society [6]. It was designed by researchers at the Institute of Social Science Survey at Peking University. The baseline survey, which was conducted in 2010, sampled in 25 provinces which encompass about 95% of the population. Follow-up surveys were done in 2012, 2014, 2016, and 2018. Note that the CFPS conducted interviews in three counties in Hubei. Most interviews (77%) were completed in July or August. In this study, we make use of the 2014, 2016, and 2018 waves, analyzing data from persons who were (1) aged 16 or older and (2) residing in one of the 25 provinces sampled at baseline. For each wave, the size of our estimation sample exceeds 30,000.

The CFPS contains a module on health. Respondents are asked whether they had a doctor-diagnosed chronic disease in the past six months. If so, the name of up to two diseases is recorded verbatim. CFPS staff subsequently categorize the verbatim responses into structured diagnosis codes. We focus on lung disease and nine other broad types of disease including cancer, diabetes and metabolic disease, psychiatric and nervous system disease, eye disease, heart disease, digestive disease, genitourinary disease, musculoskeletal disease, and physical injury. It is generally uninformative to examine individual diagnosis codes due to low incidence across provinces.

Three measures of lung disease are constructed. The first two make use of specific diagnosis codes to limit to potentially serious infections. The codes indicating sore throat, common cold, influenza, and asthma are excluded, while the codes indicating acute upper respiratory tract infection, pneumonia, emphysema, other chronic obstructive pulmonary disease, and other diseases of the respiratory system are included. The most common code is "acute upper respiratory tract infection." The first measure ("lung disease") includes cases of respondents who reported they were diagnosed under these codes. The second measure ("lung disease and hospitalized") includes cases of "lung disease" for those respondents who also reported they were hospitalized in the past year. The third measure ("bronchitis and hospitalized") makes use of a direct question asked only in 2018. It includes cases of bronchitis in the past six months for those respondents who also reported they were hospitalized in the past year. Individuals who were hospitalized were not necessarily hospitalized for a lung disease. However, those who had any diagnosis code were about 4 times more likely to report hospitalization than those who did not have a diagnosis code.

For each disease, we calculate the incidence in 2014, 2016, and 2018. Incidence is the weighted percentage of respondents who were diagnosed with the disease in the past six months. We use individual-level cross-sectional weights provided by the CFPS. Incidence is broken down by province or region. Some provinces have relatively small sample sizes, so they are grouped together with contiguous provinces. The tables display the incidence in 2018; the sum of the incidence in 2014 and 2016; the ratio of the two percentages; and the rank of the ratio from largest to smallest.

### CHARLS

The China Health and Retirement Longitudinal Study (CHARLS) is a large-scale longitudinal survey focused on older adults [7]. It was designed and funded through a collaboration between China and the United States and is often considered a sister study of the Health and Retirement Study (HRS) in the U.S. The baseline survey, which was conducted in 2011, sampled in 28 provinces which encompass about 98% of the population. Follow-up surveys were done in 2013, 2015, and 2018. Note that CHARLS conducted interviews in four counties in Hubei. None of them included Wuhan. Nearly all interviews (99%) were completed in July or August. In this study, we make use of the 2018 wave, analyzing data from persons who were (1) aged 45 or older and (2) residing in one of 27 provinces sampled at baseline (all but Xinjiang, where a multi-faceted policy initiative implemented in 2017 may cloud the results). The size of our estimation sample is almost 20,000.

CHARLS contains a module on health. Respondents are asked, one by one, whether they have been diagnosed with 14 types of chronic disease. If so, they are asked in which year they were diagnosed. The types of disease include hypertension, dyslipidemia, diabetes and metabolic disease, cancer, lung disease, liver disease, heart disease, stroke, kidney disease, digestive disease, psychiatric and nervous system disease, memory-related disease, musculoskeletal disease, and asthma. Unlike the CFPS, CHARLS does not provide diagnosis codes or any other details about the specific conditions diagnosed.

Two measures of lung disease are constructed. The first measure ("lung disease") includes cases of respondents who reported they were diagnosed with lung disease. The second ("lung disease and hospitalized") includes cases of "lung disease" for those respondents who also reported they were hospitalized in the past year. Individuals who were hospitalized were not necessarily hospitalized for a lung disease. However, those who had one of the 14 diseases were about 2.3 times more likely to report hospitalization than those who did not have any of the 14 diseases.

For each disease (except asthma which has an extremely low number of cases), we calculate the incidence in 2015–2017 and 2018. Incidence is the weighted percentage of respondents who were diagnosed with the disease. We use individual-level weights with response adjustment provided by CHARLS. Incidence is broken down by province or region. Some provinces have relatively small sample sizes, so they are grouped together with contiguous provinces. The tables display the incidence in 2018; the sum of the incidence in 2015, 2016, and 2017; the ratio of the two percentages; and the rank of the ratio from largest to smallest.

## Results

### Evidence from CFPS

Table 1 displays estimates of the incidence of lung disease by province and region. For each of the measures, the province of Hubei had the highest incidence in 2018 and also the highest growth rate between 2014/16 and 2018. Take the first measure. 1.35% of respondents from Hubei reported a diagnosis of lung disease in the past six months in the 2014 or 2016 survey waves, while 2.32% reported a diagnosis of lung disease in the past six months in the 2018 survey wave. Incidence of lung disease in 2018 was almost double the national average, which is calculated using the sample and displayed in the table. As for the second measure, 0.35% of respondents from Hubei reported hospitalization and a diagnosis of lung disease in 2014 or 2016, while 1.14% reported hospitalization and a diagnosis of lung disease in 2018. Incidence of lung disease and hospitalization in 2018 was more than double the national average. Likewise, incidence of bronchitis and hospitalization in Hubei was highest among the provinces and regions at 3.76% in 2018.

**Table 1:** Diagnosis of lung disease in past 6 months (CFPS).

						Lung disease					Bronchitis	
	Lung disease					and hospitalized					and hospitalized	
	2018	2014/16	ratio	rank	p-value	2018	2014/16	ratio	rank	p-value	2018	p-value
	%	%				%	%				%	
Hubei	2.32	1.35	1.72	1		1.14	0.35	3.23	1		3.76	
Gansu	1.25	0.78	1.60	2	0.191	0.34	0.23	1.47	3	0.070	1.73	0.007
Yunnan-Guizhou	1.05	1.08	0.98	3	0.031	0.60	0.62	0.97	8	0.030	2.47	0.020
Guangdong-Guangxi	1.18	1.36	0.87	4	0.034	0.22	0.37	0.59	12	0.003	1.00	0.000
Fujian-Jiangxi	1.78	2.10	0.85	5	0.102	1.10	0.55	1.98	2	0.576	1.48	0.000
Beijing-Tianjin-Hebei	0.88	1.04	0.85	6	0.013	0.22	0.16	1.40	5	0.008	0.68	0.000
Henan	1.04	1.31	0.79	7	0.024	0.59	0.57	1.04	7	0.041	1.56	0.000
Jiangsu-Shanghai-Zhejiang	1.21	1.62	0.75	8	0.030	0.30	0.21	1.41	4	0.014	0.91	0.000
Anhui	1.38	1.96	0.71	9	0.062	0.20	0.91	0.22	14	0.001	1.48	0.001
Sichuan-Chongqing	2.05	2.97	0.69	10	0.053	1.06	1.55	0.68	10	0.042	3.31	0.419
Heilongjiang-Jilin-Liaoning	1.05	1.54	0.68	11	0.008	0.47	0.51	0.91	9	0.013	2.00	0.001
Shaanxi-Shanxi	1.38	2.20	0.62	12	0.010	0.37	0.63	0.60	11	0.004	1.25	0.000
Shandong	0.53	1.10	0.48	13	0.002	0.26	0.55	0.47	13	0.003	0.91	0.000
Hunan	1.03	2.40	0.43	14	0.002	0.68	0.54	1.27	6	0.126	2.00	0.004
China (national level)	1.21	1.62	0.75			0.49	0.55	0.89			1.65	

NOTE

1. Cross-sectional sample weights provided by CFPS are used.

2. The columns labeled "ratio" are the weighted percentage in 2018 divided by the weighted percentage in 2014/16.

3. Rows are sorted by the percentage change in lung disease between 2014/16 and 2018.

4. P-values correspond to tests of the difference between Hubei and other provinces in the difference in outcomes between 2014/16 and 2018. To do so, outcomes are regressed on a binary indicator for 2018, binary indicators for province, and binary indicators for the interaction between province and 2018.

Table 2 displays estimates of the incidence of nine other types of disease by province and region. Hubei does not appear to exhibit notable growth between 2014/16 and 2018 in any of the types of disease, with the exception of psychiatric and nervous system disease. 0.82% of respondents from Hubei reported a diagnosis of psychiatric and nervous system disease in 2014 or 2016, while 3.07% reported a diagnosis in 2018. The ratio of incidence between 2018 and 2014/16 is the highest ratio in the table for any disease. Unfortunately, it is not insightful to examine individual diagnosis codes for psychiatric and nervous system disease. The most common code, by far, is "other."

**Table 2:** Diagnosis of disease in past 6 months (CFPS).

	Cancer				Diabetes and metabolic disease				Psychiatric and nervous system disease			
	2018	2014/16	ratio	rank	2018	2014/16	ratio	rank	2018	2014/16	ratio	rank
	%	%			%	%			%	%		
Heilongjiang-Jilin-Liaoning	0.31	0.84	0.37	11	2.45	4.54	0.54	8	0.29	1.40	0.21	14
Beijing-Tianjin-Hebei	0.25	0.54	0.47	8	2.96	5.73	0.52	10	0.39	1.55	0.25	13
Shandong	0.32	0.36	0.88	4	2.48	3.10	0.80	2	0.40	0.75	0.53	6
Anhui	0.30	1.89	0.16	14	2.49	4.30	0.58	6	0.45	0.46	0.98	3
Henan	0.27	0.57	0.47	9	2.16	3.77	0.57	7	0.37	0.75	0.50	7
Jiangsu-Shanghai-Zhejiang	0.42	0.82	0.51	7	3.35	4.83	0.69	4	0.33	0.68	0.49	8
Fujian-Jiangxi	0.70	1.60	0.44	10	2.57	2.94	0.88	1	0.87	0.61	1.42	2
Guangdong-Guangxi	0.23	0.39	0.60	6	2.08	2.70	0.77	3	0.22	0.63	0.34	10
Shaanxi-Shanxi	0.08	0.33	0.25	13	2.02	5.56	0.36	12	0.49	0.70	0.70	5
Gansu	0.35	0.49	0.72	5	1.48	4.49	0.33	13	0.37	0.75	0.48	9
Sichuan-Chongqing	0.44	0.24	1.82	1	1.53	2.86	0.54	9	0.33	1.00	0.33	11
Hubei	0.46	0.49	0.95	3	6.73	11.33	0.59	5	3.07	0.82	3.75	1
Hunan	0.39	1.16	0.34	12	1.77	5.94	0.30	14	0.78	1.06	0.73	4
Yunnan-Guizhou	0.33	0.25	1.32	2	1.00	2.69	0.37	11	0.13	0.41	0.33	12
	Eye disease				Heart disease				Digestive disease			
	2018	2014/16	ratio	rank	2018	2014/16	ratio	rank	2018	2014/16	ratio	rank
	%	%			%	%			%	%		
Heilongjiang-Jilin-Liaoning	0.73	0.76	0.96	4	8.52	20.16	0.42	11	3.00	6.68	0.45	7
Beijing-Tianjin-Hebei	0.16	0.54	0.30	12	7.78	15.67	0.50	7	2.90	6.84	0.42	10
Shandong	0.20	0.00		1	7.55	10.84	0.70	1	2.27	5.34	0.43	8
Anhui	0.30	0.12	2.55	2	10.45	18.54	0.56	3	3.27	7.76	0.42	11
Henan	0.23	0.59	0.40	10	9.33	15.74	0.59	2	2.83	6.25	0.45	6
Jiangsu-Shanghai-Zhejiang	0.19	0.41	0.45	8	5.48	11.13	0.49	8	2.39	6.06	0.39	13
Fujian-Jiangxi	0.09	0.08	1.07	3	6.44	11.60	0.55	4	3.58	7.33	0.49	3
Guangdong-Guangxi	0.16	0.21	0.77	6	5.27	11.14	0.47	9	1.83	3.87	0.47	5
Shaanxi-Shanxi	0.37	0.57	0.65	7	8.50	19.81	0.43	10	2.52	6.14	0.41	12
Gansu	0.13	0.50	0.26	13	8.23	15.96	0.52	6	5.02	10.42	0.48	4
Sichuan-Chongqing	0.19	0.45	0.42	9	3.99	9.83	0.41	13	4.03	7.24	0.56	1
Hubei	0.00	1.76	0.00	14	8.75	20.74	0.42	12	2.52	9.31	0.27	14
Hunan	0.36	0.40	0.90	5	7.67	20.68	0.37	14	3.85	7.72	0.50	2
Yunnan-Guizhou	0.08	0.20	0.37	11	3.69	6.99	0.53	5	2.27	5.37	0.42	9
	Genitourinary disease				Musculoskeletal disease				Physical injury			
	2018	2014/16	ratio	rank	2018	2014/16	ratio	rank	2018	2014/16	ratio	rank
	%	%			%	%			%	%		
Heilongjiang-Jilin-Liaoning	0.54	2.82	0.19	13	3.28	6.92	0.47	5	0.29	0.36	0.81	6
Beijing-Tianjin-Hebei	1.21	1.67	0.73	1	2.23	6.21	0.36	8	0.19	0.17	1.11	3
Shandong	0.18	1.14	0.16	14	2.99	3.75	0.80	1	0.16	0.55	0.29	12
Anhui	1.11	3.09	0.36	10	3.45	7.69	0.45	6	0.00	0.16	0.00	14
Henan	0.82	1.79	0.46	7	2.31	6.81	0.34	10	0.19	0.42	0.45	10
Jiangsu-Shanghai-Zhejiang	1.02	2.57	0.40	9	1.67	4.70	0.35	9	0.47	0.29	1.66	1
Fujian-Jiangxi	1.89	3.03	0.62	2	4.26	8.18	0.52	2	0.08	0.54	0.15	13
Guangdong-Guangxi	0.63	1.35	0.47	6	1.79	6.16	0.29	12	0.05	0.10	0.49	9
Shaanxi-Shanxi	0.54	1.56	0.35	11	2.82	5.85	0.48	4	0.14	0.22	0.64	7
Gansu	2.37	4.97	0.48	5	4.67	9.54	0.49	3	0.18	0.31	0.57	8
Sichuan-Chongqing	1.36	2.52	0.54	3	2.69	9.85	0.27	13	0.22	0.25	0.89	5
Hubei	2.12	4.76	0.44	8	2.81	9.15	0.31	11	0.11	0.25	0.42	11
Hunan	1.78	3.32	0.54	4	4.59	11.53	0.40	7	0.26	0.22	1.17	2
Yunnan-Guizhou	0.62	2.39	0.26	12	1.33	7.48	0.18	14	0.27	0.25	1.05	4

NOTE

1. Cross-sectional sample weights provided by CFPS are used.

2. The columns labeled "ratio" are the weighted percentage in 2018 divided by the weighted percentage in 2014/16.

**Fig 1:**
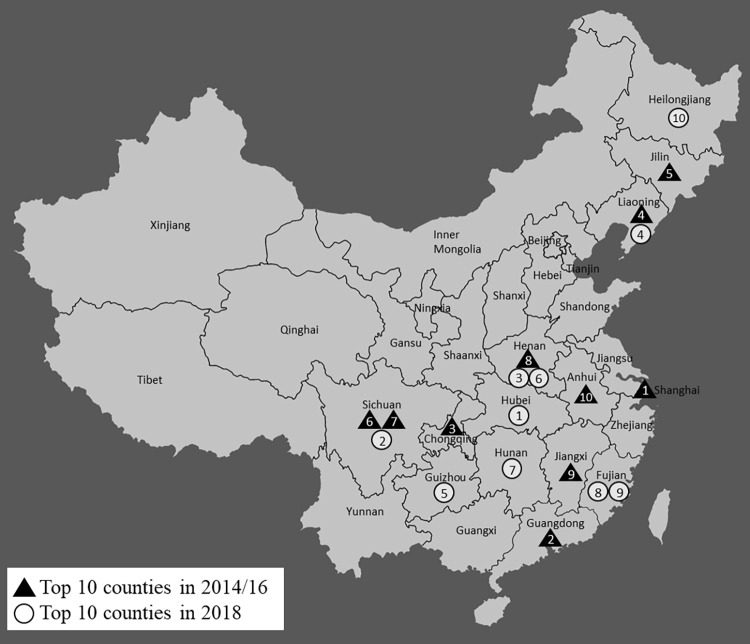
Places with the highest incidence of lung disease and hospitalization (CFPS).

Fig 1 illustrates some of the results geographically. To do so, the second measure of lung disease is calculated by county, and the top 10 counties in 2018 and in 2014/16 are identified. Altogether, there are 153 counties in our estimation sample. Only the provinces to which the counties belong, not their exact locations, are depicted in the map to protect the confidentiality of survey respondents. The rank (from 1 to 10) is labeled on the points in the map. In sum, the map shows that the location of places with the highest incidence of lung disease shifted toward central China including Hubei and neighboring provinces. Note that 2 of 3 sampled counties in Hubei are among the top 25 of 153 total counties.

### Evidence from CHARLS

Table 3 displays estimates of the incidence of lung disease by province and region. For each measure, Hubei had the highest incidence in 2018 and also the highest growth rate between 2015–17 and 2018. 2.16% of respondents from Hubei reported a diagnosis of lung disease during the 2015–2017 period, while 1.93% reported a diagnosis of lung disease during the year 2018. Incidence of lung disease in 2018 was triple the national average, which is calculated using the sample and displayed in the table. Moreover, the rate of lung disease and hospitalization was, by far, the highest among the provinces and regions.

**Table 3:** Diagnosis of lung disease by year (CHARLS).

						Lung disease	
	Lung disease					and hospitalized	
	2018	2015–17	ratio	rank	p-value	2018	p-value
	%	%				%	
Hubei	1.93	2.16	0.90	1		1.11	
Hunan	1.08	1.40	0.77	2	0.918	0.32	0.013
Shaanxi-Shanxi	0.88	2.01	0.44	3	0.331	0.43	0.028
Shandong	0.49	1.39	0.35	4	0.454	0.26	0.005
Heilongjiang-Jilin-Liaoning	0.61	1.73	0.35	5	0.322	0.16	0.002
Sichuan-Chongqing	0.93	3.10	0.30	6	0.029	0.47	0.032
Inner Mongolia	0.77	2.71	0.29	7	0.084	0.70	0.216
Gansu-Qinghai	0.99	3.78	0.26	8	0.015	0.41	0.046
Anhui	0.45	1.81	0.25	9	0.263	0.00	0.001
Guangdong-Guangxi	0.65	2.68	0.24	10	0.035	0.49	0.030
Fujian-Jiangxi	0.44	2.11	0.21	11	0.105	0.08	0.001
Yunnan-Guizhou	0.73	4.11	0.18	12	0.000	0.51	0.047
Henan	0.40	2.27	0.18	13	0.072	0.16	0.002
Jiangsu-Shanghai-Zhejiang	0.20	1.22	0.17	14	0.359	0.03	0.000
Beijing-Tianjin-Hebei	0.32	2.23	0.14	15	0.078	0.18	0.003
China (national level)	0.65	2.27	0.28			0.32	

NOTE

1. Cross-sectional sample weights provided by CHARLS are used.

2. The columns labeled "ratio" are the weighted percentage in 2018 divided by the weighted percentage in 2015–17.

3. Rows are sorted by the percentage change in lung disease between 2015–17 and 2018.

4. P-values correspond to tests of the difference between Hubei and other provinces in the difference in outcomes between 2015–17 and 2018. To do so, outcomes are regressed on a binary indicator for 2018, binary indicators for province, and binary indicators for the interaction between province and 2018.

Table 4 displays estimates of the incidence of twelve other types of disease by province and region. Hubei does not exhibit notable growth between 2015–17 and 2018 in any of the types of disease, with the exception of psychiatric and nervous system disease and digestive disease. For psychiatric and nervous system disease, Hubei had the second highest incidence in 2018 and the third highest growth from 2015–17 to 2018. For digestive disease, the province had the highest incidence in 2018 as well as the highest growth from 2015–17 to 2018. It may be useful to know that in the CFPS, the most common diagnosis within the category of digestive disease is gastroenteritis, which is typified by diarrhea or vomiting.

**Table 4:** Diagnosis of disease by year (CHARLS).

	Hypertension				Dyslipidemia				Diabetes and metabolic disease			
	2018	2015–17	ratio	rank	2018	2015–17	ratio	rank	2018	2015–17	ratio	rank
	%	%			%	%			%	%		
Inner Mongolia	1.10	5.67	0.19	13	1.29	5.62	0.23	12	1.01	2.64	0.38	5
Heilongjiang-Jilin-Liaoning	1.61	4.56	0.35	3	1.72	4.82	0.36	4	1.29	2.23	0.58	1
Beijing-Tianjin-Hebei	1.96	5.63	0.35	4	1.41	5.45	0.26	11	0.98	3.10	0.32	9
Shandong	1.80	6.73	0.27	11	1.75	5.67	0.31	9	1.61	3.07	0.52	3
Anhui	1.98	6.84	0.29	8	1.99	6.10	0.33	6	1.57	4.26	0.37	6
Henan	1.64	5.92	0.28	9	1.56	4.58	0.34	5	0.77	3.36	0.23	14
Jiangsu-Shanghai-Zhejiang	1.29	4.80	0.27	10	1.58	4.97	0.32	7	1.23	2.17	0.57	2
Fujian-Jiangxi	2.02	6.12	0.33	5	0.99	2.62	0.38	3	0.67	3.20	0.21	15
Guangdong-Guangxi	0.83	7.00	0.12	15	0.94	7.15	0.13	15	0.81	3.47	0.23	13
Shaanxi-Shanxi	1.94	5.38	0.36	2	1.76	5.88	0.30	10	0.86	2.98	0.29	11
Gansu-Qinghai	3.39	8.64	0.39	1	2.55	5.09	0.50	1	1.04	3.33	0.31	10
Sichuan-Chongqing	1.75	6.81	0.26	12	1.92	4.34	0.44	2	1.53	3.03	0.51	4
Hubei	1.60	5.15	0.31	7	1.70	5.45	0.31	8	0.75	2.23	0.34	8
Hunan	0.89	6.14	0.14	14	1.29	5.77	0.22	13	0.85	3.38	0.25	12
Yunnan-Guizhou	2.11	6.51	0.32	6	0.68	3.15	0.22	14	0.53	1.55	0.34	7
	Cancer				Liver disease				Heart disease			
	2018	2015–17	ratio	rank	2018	2015–17	ratio	rank	2018	2015–17	ratio	rank
	%	%			%	%			%	%		
Inner Mongolia	0.17	0.05	3.80	2	1.33	1.59	0.84	4	2.25	4.49	0.50	3
Heilongjiang-Jilin-Liaoning	0.24	0.81	0.30	9	1.23	1.55	0.80	6	1.46	4.01	0.36	9
Beijing-Tianjin-Hebei	0.09	0.97	0.09	13	0.27	0.67	0.41	8	1.85	4.38	0.42	6
Shandong	0.00	0.44	0.00	14	0.37	1.09	0.34	10	2.12	4.97	0.43	5
Anhui	0.18	0.40	0.45	6	0.38	1.15	0.33	11	1.72	3.85	0.45	4
Henan	0.13	0.69	0.19	11	0.73	0.90	0.81	5	0.67	4.25	0.16	12
Jiangsu-Shanghai-Zhejiang	0.62	0.78	0.80	3	0.12	0.49	0.24	12	0.20	1.42	0.14	13
Fujian-Jiangxi	0.13	0.53	0.24	10	0.56	1.53	0.37	9	0.31	2.63	0.12	15
Guangdong-Guangxi	0.00	0.69	0.00	15	0.03	2.08	0.01	15	0.34	0.82	0.41	7
Shaanxi-Shanxi	0.27	0.87	0.31	8	0.18	1.12	0.16	13	1.54	2.99	0.51	2
Gansu-Qinghai	0.48	0.10	4.83	1	1.52	1.16	1.30	1	1.83	3.29	0.56	1
Sichuan-Chongqing	0.19	0.58	0.33	7	0.71	1.60	0.44	7	0.93	2.66	0.35	10
Hubei	0.10	0.68	0.14	12	0.35	2.22	0.16	14	0.46	3.59	0.13	14
Hunan	0.34	0.62	0.54	4	0.54	0.49	1.10	2	1.25	3.32	0.38	8
Yunnan-Guizhou	0.13	0.29	0.46	5	0.86	0.97	0.89	3	0.48	2.20	0.22	11
	Stroke				Kidney disease				Digestive disease			
	2018	2015–17	ratio	rank	2018	2015–17	ratio	rank	2018	2015–17	ratio	rank
	%	%			%	%			%	%		
Inner Mongolia	0.41	1.88	0.22	8	1.50	2.37	0.63	6	0.88	6.74	0.13	15
Heilongjiang-Jilin-Liaoning	0.32	3.70	0.09	14	1.50	1.99	0.76	2	1.28	2.44	0.53	2
Beijing-Tianjin-Hebei	0.10	2.88	0.03	15	0.29	1.16	0.25	12	0.51	2.55	0.20	12
Shandong	0.51	0.69	0.74	1	0.30	0.76	0.39	9	0.60	2.24	0.27	8
Anhui	0.98	4.58	0.21	9	0.26	2.00	0.13	13	1.23	4.24	0.29	7
Henan	0.97	2.87	0.34	4	0.80	1.48	0.54	7	1.07	2.67	0.40	3
Jiangsu-Shanghai-Zhejiang	0.47	1.62	0.29	7	0.68	1.00	0.68	4	0.53	2.38	0.22	11
Fujian-Jiangxi	0.47	1.07	0.44	3	0.66	1.44	0.46	8	0.51	3.12	0.16	14
Guangdong-Guangxi	0.17	1.57	0.11	13	0.16	3.73	0.04	15	0.54	2.88	0.19	13
Shaanxi-Shanxi	0.51	2.89	0.18	10	0.93	1.44	0.64	5	1.13	2.86	0.40	4
Gansu-Qinghai	0.27	2.03	0.13	12	1.63	1.92	0.85	1	1.37	4.07	0.34	5
Sichuan-Chongqing	0.51	1.69	0.30	6	0.88	2.52	0.35	10	1.02	3.30	0.31	6
Hubei	0.36	2.40	0.15	11	0.12	1.68	0.07	14	1.42	2.43	0.58	1
Hunan	1.25	2.80	0.45	2	0.91	1.31	0.69	3	0.48	2.11	0.23	10
Yunnan-Guizhou	0.51	1.63	0.31	5	0.87	2.62	0.33	11	0.88	3.78	0.23	9
	Psychiatric and nervous system disease				Memory-related disease				Musculoskeletal disease			
	2018	2015–17	ratio	rank	2018	2015–17	ratio	rank	2018	2015–17	ratio	rank
	%	%			%	%			%	%		
Inner Mongolia	0.18	1.49	0.12	11	0.25	2.39	0.11	14	0.35	2.78	0.13	14
Heilongjiang-Jilin-Liaoning	0.19	0.46	0.42	5	0.60	1.02	0.58	3	0.88	1.59	0.56	1
Beijing-Tianjin-Hebei	0.16	0.29	0.54	4	0.20	1.04	0.19	11	0.62	3.05	0.20	11
Shandong	0.06	0.47	0.12	12	1.18	1.00	1.17	2	1.09	2.32	0.47	3
Anhui	0.38	0.44	0.87	2	0.48	0.96	0.50	5	0.77	3.49	0.22	10
Henan	0.07	0.60	0.12	13	0.43	1.67	0.26	10	0.91	1.88	0.48	2
Jiangsu-Shanghai-Zhejiang	0.07	0.35	0.19	8	0.21	0.44	0.48	6	0.25	1.38	0.18	12
Fujian-Jiangxi	0.08	0.52	0.15	10	0.04	0.64	0.07	15	0.15	2.61	0.06	15
Guangdong-Guangxi	0.09	0.36	0.25	7	0.09	0.82	0.11	13	1.43	4.44	0.32	5
Shaanxi-Shanxi	0.07	0.72	0.10	14	0.22	1.17	0.19	12	0.45	2.78	0.16	13
Gansu-Qinghai	0.11	1.30	0.09	15	0.12	0.42	0.29	8	0.57	1.95	0.29	7
Sichuan-Chongqing	0.19	1.12	0.17	9	0.45	1.06	0.42	7	1.02	2.17	0.47	4
Hubei	0.54	0.92	0.58	3	0.57	1.14	0.51	4	1.26	4.11	0.31	6
Hunan	0.70	0.68	1.03	1	0.70	0.42	1.67	1	0.62	2.63	0.24	9
Yunnan-Guizhou	0.21	0.54	0.40	6	0.18	0.69	0.27	9	0.94	3.81	0.25	8

NOTE: 1. Cross-sectional sample weights provided by CHARLS are used.

2. The columns labeled "ratio" are the weighted percentage in 2018 divided by the weighted percentage in 2015–17.

**Fig 2:**
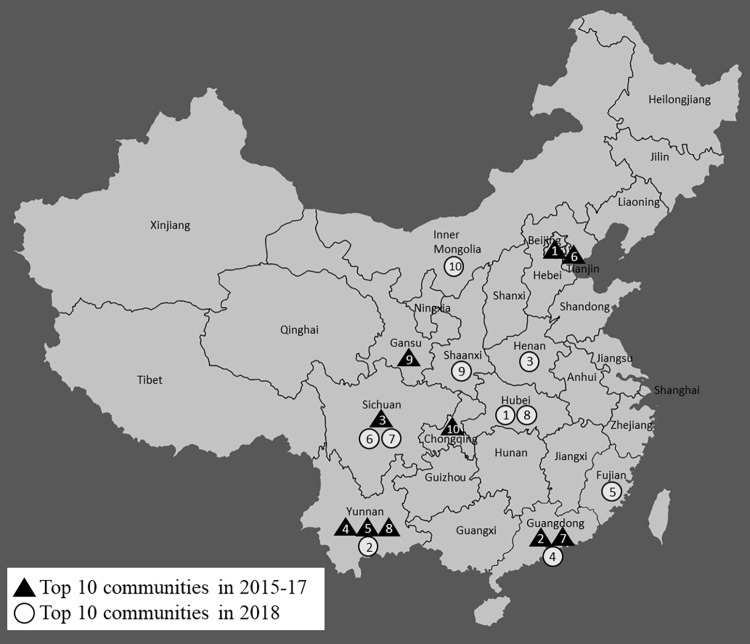
Places with the highest incidence of lung disease (CHARLS).

Fig 2 illustrates some of the results geographically. To do so, the first measure of lung disease is calculated by village/urban community, and the top 10 communities in 2018 and in 2015–17 are identified. Altogether, there are 446 communities in our estimation sample. Only the provinces to which the communities belong, not their exact locations, are depicted in the map to protect the confidentiality of survey respondents. The rank (from 1 to 10) is labeled on the points in the map. In sum, the map shows that the location of places with the highest incidence of lung disease shifted toward central China including Hubei and neighboring provinces. Note that 5 of 12 sampled communities in Hubei are among the top 25 of 446 total communities.

### Further evidence

Table 5 examines the incidence of lung disease by province and year, and Table 6 examines the incidence of psychiatric and nervous system disease by province and year. Though more noisy, the patterns are roughly similar to the previous results.

**Table 5:** Diagnosis of lung disease by province and year.

	Statistics from CFPS							Statistics from CHARLS				
				Lung disease			Bronchitis					Lung disease
	Lung disease			and hospitalized			and hosp.	Lung disease				and hosp.
	2018	2016	2014	2018	2016	2014	2018	2018	2017	2016	2015	2018
	%	%	%	%	%	%	%	%	%	%	%	%
Beijing	0.89	0.75	2.18	0.00	0.00	0.14	0.00	0.00	2.97	0.00	0.00	0.00
Tianjin	2.03	1.52	0.23	0.00	0.00	0.00	0.00	0.00	3.34	0.00	0.87	0.00
Hebei	0.73	0.12	0.56	0.27	0.02	0.15	0.85	0.44	0.91	0.41	0.36	0.24
Shanxi	1.30	1.50	1.01	0.50	0.50	0.15	1.59	0.35	0.87	0.45	0.19	0.00
Liaoning	0.89	0.39	0.58	0.24	0.11	0.21	0.95	0.50	0.66	0.55	0.41	0.00
Jilin	1.11	0.70	1.66	0.22	0.55	0.58	4.11	0.48	0.90	0.45	0.00	0.32
Heilongjiang	1.14	0.12	1.34	0.76	0.00	0.31	1.62	0.85	1.65	0.51	0.00	0.23
Shanghai	1.46	1.43	0.95	0.23	0.07	0.27	1.47	0.00	1.24	0.00	0.00	0.00
Jiangsu	1.31	1.11	0.46	0.44	0.00	0.11	1.23	0.10	0.70	0.51	0.23	0.00
Zhejiang	0.95	0.30	0.87	0.22	0.00	0.23	0.28	0.36	0.61	0.14	0.24	0.06
Anhui	1.38	1.12	0.84	0.20	0.46	0.45	1.48	0.45	0.75	0.43	0.63	0.00
Fujian	1.70	0.22	1.35	1.70	0.22	0.08	1.48	0.43	1.34	0.52	0.18	0.00
Jiangxi	1.84	1.10	1.40	0.55	0.48	0.30	1.48	0.45	1.00	0.27	0.89	0.12
Shandong	0.53	0.47	0.62	0.26	0.25	0.30	0.91	0.49	0.85	0.42	0.13	0.26
Henan	1.04	0.49	0.82	0.59	0.25	0.32	1.56	0.40	1.05	0.58	0.65	0.16
Hubei	2.32	0.17	1.19	1.14	0.17	0.19	3.76	1.93	1.42	0.61	0.12	1.11
Hunan	1.03	0.85	1.56	0.68	0.15	0.39	2.00	1.08	0.79	0.25	0.37	0.32
Guangdong	1.14	0.88	0.64	0.29	0.33	0.17	0.87	0.65	2.14	0.47	0.32	0.62
Guangxi	1.32	0.41	0.48	0.00	0.00	0.00	1.43	0.65	0.45	1.11	0.48	0.15
Chongqing	2.36	1.74	1.95	0.87	0.77	1.00	3.78	0.88	2.23	2.42	0.47	0.30
Sichuan	2.01	0.79	2.09	1.09	0.59	0.93	3.24	0.94	1.63	0.51	0.59	0.50
Guizhou	1.23	0.15	0.89	0.59	0.03	0.61	2.74	0.53	0.00	0.46	0.45	0.54
Yunnan	0.84	0.20	0.92	0.62	0.09	0.53	2.15	0.76	2.16	1.25	1.24	0.51
Shaanxi	1.54	0.69	0.90	0.12	0.21	0.41	0.56	1.28	1.52	0.75	0.12	0.76
Gansu	1.25	0.23	0.55	0.34	0.03	0.20	1.73	1.06	2.31	1.56	0.16	0.32
Qinghai								0.74	1.39	0.00	1.40	0.74
Inner Mongolia								0.77	0.77	0.71	1.23	0.70

NOTE: Cross-sectional sample weights provided by CFPS and CHARLS are used.

**Table 6:** Diagnosis of psychiatric and nervous system disease by province and year.

	Statistics from CFPS			Statistics from CHARLS			
	Psychiatric and nervous			Psychiatric and nervous			
	system disease			system disease			
	2018	2016	2014	2018	2017	2016	2015
	%	%	%	%	%	%	%
Beijing	0.00	0.37	0.42	0.00	0.00	0.00	0.00
Tianjin	0.65	0.47	0.00	0.00	0.00	0.00	0.00
Hebei	0.40	1.02	0.79	0.21	0.05	0.29	0.05
Shanxi	0.70	0.44	0.36	0.16	0.35	0.00	0.00
Liaoning	0.17	0.37	0.41	0.46	0.30	0.58	0.00
Jilin	0.14	0.76	0.25	0.00	0.18	0.00	0.00
Heilongjiang	0.45	1.15	0.87	0.00	0.13	0.00	0.00
Shanghai	0.24	0.50	0.28	0.00	0.00	0.00	0.00
Jiangsu	0.00	0.66	0.38	0.00	0.08	0.31	0.00
Zhejiang	0.66	0.00	0.24	0.15	0.15	0.06	0.18
Anhui	0.45	0.46	0.00	0.38	0.00	0.22	0.22
Fujian	1.32	0.96	0.00	0.00	0.19	0.00	0.00
Jiangxi	0.47	0.22	0.15	0.12	0.40	0.00	0.30
Shandong	0.40	0.61	0.13	0.06	0.14	0.20	0.13
Henan	0.37	0.54	0.22	0.07	0.22	0.11	0.26
Hubei	3.07	0.19	0.63	0.54	0.16	0.27	0.49
Hunan	0.78	0.85	0.21	0.70	0.45	0.23	0.00
Guangdong	0.27	0.49	0.26	0.00	0.10	0.00	0.08
Guangxi	0.06	0.32	0.00	0.32	0.55	0.10	0.18
Chongqing	0.56	0.42	0.37	0.00	0.36	0.00	0.22
Sichuan	0.30	0.61	0.42	0.23	0.51	0.49	0.22
Guizhou	0.00	0.06	0.00	0.53	0.00	0.00	0.52
Yunnan	0.29	0.84	0.06	0.16	0.16	0.12	0.25
Shaanxi	0.07	0.35	0.13	0.00	0.40	0.60	0.00
Gansu	0.37	0.40	0.35	0.14	0.12	0.81	0.16
Qinghai				0.00	1.08	0.00	1.07
Inner Mongolia				0.18	0.17	0.81	0.51

NOTE: Cross-sectional sample weights provided by CFPS and CHARLS are used.

Table 7 breaks down the incidence of lung disease by age. For the CFPS, respondents aged 16–44 in 2018 are compared with those aged 45–70 in 2018. Lung disease increased rapidly in Hubei for both age groups. However, growth was larger for respondents aged 45–70. For CHARLS, respondents aged 45–70 in 2018 are compared with those above age 70 in 2018. Note that respondents above age 70 make up less than 25% of the sample, and statistics for this group might be less reliable because of attrition due to mortality and other reasons. Like the CFPS, growth was relatively larger for respondents aged 45–70 in 2018.

**Table 7:** Diagnosis of lung disease by age.

	Statistics from CFPS							
	Age 16–44 in 2018				Age 45–70 in 2018			
	2018	2014/16	ratio	rank	2018	2014/16	ratio	rank
	%	%			%	%		
Heilongjiang-Jilin-Liaoning	0.50	0.77	0.65	9	1.29	1.35	0.96	8
Beijing-Tianjin-Hebei	0.77	0.71	1.08	4	1.00	0.80	1.24	4
Shandong	0.00	0.50	0.00	14	0.70	1.53	0.46	14
Anhui	0.61	0.84	0.73	8	2.01	3.03	0.66	12
Henan	0.32	0.82	0.39	12	1.42	1.63	0.87	9
Jiangsu-Shanghai-Zhejiang	1.31	1.66	0.79	7	1.03	0.90	1.14	5
Fujian-Jiangxi	2.04	2.12	0.96	5	1.77	2.20	0.81	11
Guangdong-Guangxi	1.02	1.09	0.94	6	1.35	1.32	1.02	6
Shaanxi-Shanxi	0.54	1.20	0.45	10	2.22	2.22	1.00	7
Gansu	0.80	0.41	1.95	1	1.72	1.02	1.68	2
Sichuan-Chongqing	0.45	1.29	0.35	13	2.75	3.27	0.84	10
Hubei	1.02	0.73	1.39	3	2.86	1.58	1.81	1
Hunan	1.46	3.48	0.42	11	0.91	1.68	0.54	13
Yunnan-Guizhou	0.94	0.55	1.70	2	1.13	0.76	1.50	3
	Statistics from CHARLS							
	Age 45–70 in 2018				Age 70+ in 2018			
	2018	2015–17	ratio	rank	2018	2015–17	ratio	rank
	%	%			%	%		
Inner Mongolia	0.55	2.78	0.20	11	1.62	2.43	0.66	3
Heilongjiang-Jilin-Liaoning	0.77	1.71	0.45	3	0.00	1.78	0.00	13
Beijing-Tianjin-Hebei	0.42	2.49	0.17	15	0.00	1.37	0.00	14
Shandong	0.28	1.41	0.20	10	1.30	1.32	0.98	1
Anhui	0.29	1.49	0.20	12	0.94	2.80	0.34	5
Henan	0.46	2.55	0.18	14	0.16	1.19	0.13	9
Jiangsu-Shanghai-Zhejiang	0.24	1.18	0.20	9	0.10	1.36	0.08	12
Fujian-Jiangxi	0.52	2.20	0.24	8	0.20	1.83	0.11	10
Guangdong-Guangxi	0.59	2.33	0.25	7	0.93	4.14	0.22	8
Shaanxi-Shanxi	0.67	2.01	0.34	4	1.53	2.01	0.76	2
Gansu-Qinghai	1.05	4.04	0.26	6	0.77	2.67	0.29	7
Sichuan-Chongqing	0.81	2.71	0.30	5	1.27	4.18	0.30	6
Hubei	2.16	1.97	1.10	2	1.21	2.76	0.44	4
Hunan	1.39	1.24	1.12	1	0.00	1.96	0.00	15
Yunnan-Guizhou	0.84	4.31	0.19	13	0.34	3.42	0.10	11

NOTE: Cross-sectional sample weights provided by CFPS and CHARLS are used.

Table 8 breaks down the incidence of lung disease by smoking status. For the CFPS, incidence in Hubei grew more among respondents who had never smoked, relative to other provinces. In contrast, for CHARLS, incidence in Hubei grew faster among respondents who had ever smoked, relative to other provinces.

**Table 8:** Diagnosis of lung disease by smoking status.

	Statistics from CFPS							
	Never smoked				Ever smoked			
	2018	2014/16	ratio	rank	2018	2014/16	ratio	rank
	%	%			%	%		
Heilongjiang-Jilin-Liaoning	1.41	1.65	0.85	7	0.37	1.27	0.29	13
Beijing-Tianjin-Hebei	0.93	0.91	1.02	3	0.77	1.38	0.55	10
Shandong	0.45	1.32	0.34	14	0.80	0.23	3.48	1
Anhui	1.11	1.89	0.59	11	1.99	2.13	0.93	7
Henan	1.11	1.30	0.85	8	0.86	1.28	0.67	8
Jiangsu-Shanghai-Zhejiang	1.44	1.60	0.90	6	0.61	1.69	0.36	12
Fujian-Jiangxi	1.60	2.53	0.63	10	2.18	0.83	2.61	2
Guangdong-Guangxi	1.27	1.32	0.96	5	0.98	1.47	0.67	9
Shaanxi-Shanxi	1.81	2.41	0.75	9	0.50	1.73	0.29	14
Gansu	1.48	0.92	1.61	2	0.77	0.42	1.85	3
Sichuan-Chongqing	2.07	3.56	0.58	12	2.00	1.64	1.22	6
Hubei	2.14	1.19	1.80	1	2.81	1.73	1.62	4
Hunan	1.02	2.37	0.43	13	1.07	2.50	0.43	11
Yunnan-Guizhou	1.42	1.46	0.98	4	0.41	0.27	1.50	5
	Statistics from CHARLS							
	Never smoked				Ever smoked			
	2018	2015–17	ratio	rank	2018	2015–17	ratio	rank
	%	%			%	%		
Inner Mongolia	0.25	2.58	0.10	13	1.47	2.88	0.51	5
Heilongjiang-Jilin-Liaoning	0.65	1.65	0.40	4	0.56	1.81	0.31	7
Beijing-Tianjin-Hebei	0.00	2.66	0.00	15	0.74	1.66	0.45	6
Shandong	0.23	1.47	0.16	12	0.88	1.28	0.69	4
Anhui	0.76	1.20	0.63	3	0.00	2.68	0.00	15
Henan	0.39	1.81	0.22	8	0.42	2.98	0.14	12
Jiangsu-Shanghai-Zhejiang	0.25	1.18	0.21	9	0.14	1.28	0.11	14
Fujian-Jiangxi	0.35	1.67	0.21	10	0.58	2.72	0.21	9
Guangdong-Guangxi	0.23	3.34	0.07	14	1.31	1.66	0.79	3
Shaanxi-Shanxi	1.41	1.84	0.77	1	0.29	2.19	0.13	13
Gansu-Qinghai	1.06	3.06	0.35	7	0.91	4.70	0.19	10
Sichuan-Chongqing	0.97	2.66	0.37	6	0.88	3.76	0.23	8
Hubei	0.97	2.62	0.37	5	3.17	1.56	2.04	1
Hunan	0.81	1.22	0.66	2	1.45	1.65	0.88	2
Yunnan-Guizhou	0.74	3.86	0.19	11	0.71	4.41	0.16	11

NOTE: Cross-sectional sample weights provided by CFPS and CHARLS are used.

## Discussion

The main purpose of the paper is to describe spatiotemporal patterns of lung disease in China prior to 2019. Quantitative analysis of the CFPS and CHARLS reveals that respondents living in Hubei province reported substantial growth in lung disease in 2018 relative to respondents living in other provinces. The increase was more pronounced among respondents aged 45–70. In the CFPS and CHARLS, the incidence of psychiatric and nervous system disease also increased in Hubei during 2018. In CHARLS, the incidence of digestive disease was relatively high as well. Additionally, the mapping exercise illustrates that the location of places with the highest incidence of lung disease shifted toward central China, including Hubei, in 2018. Overall, the evidence is consistent with many possible explanations. In what follows, we offer two conjectures–not formal hypotheses–to motivate further investigation.

One conjecture is that there was an outbreak of influenza in central China. We believe this is the more likely conjecture of the two. Influenza was more severe in the 2017–2018 season relative to previous seasons [8,9], especially in central China [10,11]. Researchers from the Chinese Center for Disease Control and Prevention examined pooled data from clinically diagnosed and lab confirmed cases to show that influenza surged between 2005 and 2018 [11]. Even though a large part of the temporal increase was due to a change in the surveillance protocol in 2017, the influenza burden was especially high in central China where H1N1 was rising. If this conjecture is accurate, it implies that China was struggling with unusually high rates of lung infection in the very places where COVID would soon emerge. The conditions that increased the susceptibility to influenza also facilitated the spread of COVID. Furthermore, regional outbreaks of influenza could have masked the early spread of COVID, perhaps delaying its discovery.

Another conjecture is that COVID was circulating at low levels in the population in central China during 2018. We believe this is the less likely conjecture of the two. The evidence in the paper is consistent with the facts that the first cases of COVID were identified in Hubei and that COVID impacts lung functioning. Neurological and psychiatric diagnoses are also significantly more common among persons who had a COVID diagnosis than among persons who had an influenza diagnosis [12]. Indeed, a growing area of research concerns the mechanisms by which COVID influences brain and nervous system functioning [13,14]. If this conjecture is accurate, it casts doubt on the notion that COVID came from a laboratory in the city of Wuhan. It could simply be that the “lab leak” theory arose when COVID came to Wuhan from other parts of Hubei or central China.

All in all, this paper sheds light on spatiotemporal patterns of disease in China prior to 2019. Given the lack of epidemiological evidence publicly available, this paper makes a contribution to the literature, even though using survey data is far from ideal. Modest sample sizes necessitate geographic and temporal aggregation. Also, reported diagnoses are blurred by imperfect recall and understanding of diseases by respondents as well as imperfect categorization of diseases by survey administrators. More investigation is necessary to understand the conditions surrounding the emergence of COVID.
